# CCNI2 promotes pancreatic cancer through PI3K/AKT signaling pathway

**DOI:** 10.17305/bb.2023.9337

**Published:** 2024-04-01

**Authors:** Bingyang Hu, Wenzhi Zhang, Changsheng Zhang, Chonghui Li, Ning Zhang, Ke Pan, Xinlan Ge, Tao Wan

**Affiliations:** 1 Department of Hepatobiliary Surgery, The First Medical Center, Chinese PLA General Hospital, Chinese PLA Medical School, Haidian District, Beijing, China; 2 Department of General Surgery, Kaifeng Central Hospital, Longting District, Kaifeng, Henan Province, China

**Keywords:** Pancreatic cancer, cyclin I-like protein (CCNI2), prognosis, phenotype

## Abstract

Globally, pancreatic cancer is recognized as one of the deadliest malignancies that lacks effective targeted therapies. This study aims to explore the role of cyclin I-like protein (CCNI2), a homolog of cyclin I (CCNI), in the progression of pancreatic cancer, thereby providing a theoretical basis for its treatment. Firstly, the expression of CCNI2 in pancreatic cancer tissues was determined through immunohistochemical staining. The biological role of CCNI2 in pancreatic cancer cells was further assessed using both in vitro and in vivo loss/gain-of-function assays. Our data revealed that CCNI2 expression was abnormally elevated in pancreatic cancer, and clinically, increased CCNI2 expression generally correlated with reduced overall survival. Functionally, CCNI2 contributed to the malignant progression of pancreatic cancer by promoting the proliferation and migration of tumor cells. Consistently, in vivo experiments verified that CCNI2 knockdown impaired the tumorigenic ability of pancreatic cancer cells. Moreover, the addition of phosphatidylinositol 3-kinase (PI3K) inhibitors could partially reverse the promoting effect of CCNI2 on the malignant phenotypes of pancreatic cancer cells. CCNI2 promoted pancreatic cancer through PI3K/protein kinase B (AKT) signaling pathway, indicating its potential as a prognostic marker and therapeutic target for pancreatic cancer.

## Introduction

Pancreatic cancer is a malignant disease originating in the digestive tract [1]. It is considered one of the deadliest malignancies due to its rapid progression and high morbidity and mortality rates [1, 2]. Unfortunately, most patients with pancreatic cancer are diagnosed at an advanced stage, which eliminates the possibility of surgery as a treatment option [3]. As a result, additional treatments, such as FOLFIRINOX or gemcitabine, are used to complement the pancreatic cancer therapy [4]. Although these treatments provide some improvement in the quality of life for patients, the overall survival rate remains minimal [5]. Currently, there is a lack of effective clinical management measures for pancreatic cancer, resulting in a poor prognosis and an extremely low 5-year survival rate [1]. Given this challenging situation, it is an imperative to deeply explore the molecular mechanisms of pancreatic cancer in order to identify effective small molecular targets. This will provide a reliable theoretical basis for the treatment of this disease.

The cyclin-dependent kinase 5 (CDK5), an important kinase, requires other proteins, such as p35, p39, and cyclin I (CCNI) to activate its kinase activity [6–8]. Cyclin I-like protein (CCNI2) is considered to be a homolog of CCNI, with a higher binding affinity to CDK5 than the previously determined CDK5 activator, CCNI [9]. Specifically, CCNI2 can effectively activate CDK5 and regulate the cell cycle in the cytoplasm and membrane [9]. Furthermore, Lai et al. [10] reported that CCNI2 contributes to the malignant progression of colorectal cancer and can serve as a prognostic marker for the disease. Chen et al. [11] demonstrated that CCNI2 promotes the proliferation and migration of human gastric cancer through the transcription factor known as the hepatoma-derived growth factor (HDGF). Considering the important role of CCNI2 in cell cycle regulation and tumor cells, we were interested in determining whether CCNI2 plays a specific key role in pancreatic cancer. Therefore, we explored the role of CCNI2 in pancreatic cancer progression to provide a theoretical basis for the treatment of this disease.

In this study, we attempt to explore the role of CCNI2 in the progression of pancreatic cancer. Firstly, the correlation between CCNI2 expression and pancreatic cancer was analyzed. Moreover, the promotive role of CCNI2 in pancreatic cancer was assessed both in vitro and in vivo. As a result, CCNI2 may serve as a prognostic marker and potential therapeutic target for pancreatic cancer.

## Materials and methods

### Immunohistochemistry

The 61 tumor tissues and 55 normal tissues from pancreatic cancer patients were purchased from the Shanghai Outdo Biotech Company. These tissue samples were sealed with citric acid, dewaxed, and incubated with the primary monoclonal antibody (Anti-CCNI2, dilution: 1:200, Abcam, USA, Cat. # ab97767) at 4 ^∘^C overnight and the secondary antibody (Goat Anti-Rabbit IgG H&L horseradish peroxidase [HRP], dilution: 1:100, Abcam, USA, Cat. # ab205718) at room temperature for 3 h. Furthermore, the buffer solution with phosphate-buffered saline (PBS), serving as a blank control, was used in place of the primary antibody to stain the tissue. The tissue samples were stained with 3,3’-diaminobenzidine (DAB) and hematoxylin and scored according to the methods provided in the literature [12].

### Cell culture condition

Normal human pancreatic duct epithelial cells (HPDE6-C7) and pancreatic cancer cell lines (PANC-1, SW1990, BxPc-3) were obtained from the Cell Bank of the Chinese Academy of Sciences (Shanghai, China). All the cell lines were cultured in the Dulbecco’s Modified Eagle Medium (DMEM, GibcoBRL, Grand Island, NY, USA) supplemented with 10% fetal bovine serum (FBS, Gibco BRL) and kept at 37 ^∘^C under 5% CO_2_ atmosphere.

### Lentiviral transduction

Small hairpin RNA (shRNA) sequences targeting CCNI2 (shCCNI2-1: 5’-TACCTGCATTGCGCCACAATT-3’; shCCNI2-2: 5’-ATCTGCGACGCCTTCGAGGAA-3’; shCCNI2-3: 5’-CCTGGAAGGCGACCTGGACGA-3’) and lentivirus vectors (BR-V108) with green fluorescent protein (GFP) tags were purchased from BioRes (Shanghai, China). The target sequences (negative short hairpin control [shCtrl] and shCCNI2-1/2/3) were connected to the BR-V108 vector through T4 DNA ligase and then transduced into 2×10^5^ PANC1 and SW1990 cells at a multiplicity of infection (MOI) of 10 using Lipofectamine 3000 (Invitrogen). After 72 h, the efficiency of transfection was assessed based on the expression of the GFP and the selection of stable knockdown cell lines using purinomycin [13]. In subsequent experiments, CCNI2 expression in pancreatic cancer cells was upregulated using a similar method. Before conducting any experiments, we purified the cells transduced with the shRNA vector using fluorescence-activated cell sorting (FACS), based on their expression of the GFP.

### Quantitative PCR

Total RNA was isolated and purified from cell lines PANC-1, SW1990, BxPc-3, and HPDE6-C7 using the TRIzol reagent (Sigma-Aldrich, St. Louis, MO, USA). The quantity and quality of the extracted RNA were determined using a Nanodrop 2000/2000C spectrophotometer (Thermo Fisher Scientific, Wilmington, DE, USA). Following extraction, the RNA was reversed to synthesize the corresponding cDNA using the HiScript Q RT Supermix for qPCR (Vazyme Biotech, Shanghai, China) according to the manufacturer’s protocols. The synthetic cDNA acted as a template to implement the qPCR assays using the AceQ qPCR SYBR Green Master Mix (Vazyme, Nanjing, China) and the ABI StepOnePlus Real-Time PCR System (Applied Biosystems, Foster City, CA, USA). The glyceraldehyde 3-phosphate dehydrogenase gene (*GAPDH*) was used as a control (*GAPDH* upstream primer sequence: 5’-TGACTTCAACAGCGACACCCA-3’, downstream primer sequence: 5’-CACCCTGTTGCTGTAGCCAAA-3’) to quantify the relative mRNA expression level of CCNI2 (CCNI2 upstream primer sequence: 5’-CCAGGGAGTATGAATGAATGTT-3’, downstream primer sequence: 5’-TTGGGATAAGCCTGGGAAGTT-3’).

### Western blotting

CCNI2 protein expression in PANC-1 and SW1990 was determined using the WB analysis as previously described [14, 15]. Total cellular protein was extracted using 1× Cell Lysis Buffer (Promega, Madison, WI, USA) and protein concentrations were quantified by BCA Protein Assay Kit (Pierce, South Logan, UT, USA). Briefly, proteins were separated by 10% sodium dodecyl-sulfate polyacrylamide gel electrophoresis (SDS-PAGE) and transferred to a polyvinylidene fluoride (PVDF) membrane at 4 ^∘^C. After a 1 h block with 5% bovine serum albumin (BSA) in Tris-buffered saline and Tween 20 (TBST) at room temperature, the membrane was incubated overnight at 4 ^∘^C with primary antibodies, such as CCNI2 (1:1000, Abcam, Cat. # ab97767), AKT (1:1000, CST, Cat. # 4685), p-AKT (1:1000, Bioss, Cat. # BS5193R), CDK5 (1:1000, Abcam, Cat. # ab40773), CDK6 (1:1000, Abcam, Cat. # ab15127), MAPK9 (1:1000, Abcam, Cat. # ab76125), PIK3CA (1:1000, Abcam, Cat. # ab40776), and GAPDH (1:1000, Bioworld, Cat. # AP0063). Subsequently, the membranes were washed and incubated for 2 h at room temperature with HRP-conjugated secondary antibodies, specifically Goat Anti-Rabbit IgG (1: 3000, Beyotime, Cat. # A0208). Finally, the blots were imaged using the enhanced chemiluminescence ECL+PlusTM western blotting detection system (Amersham Pharmacia Biotech, Arlington Heights, IL, USA) and a luminescent image analyzer.

### MTT assay

Cell proliferation was evaluated using the 3-(4,5-dimethyl-2-thiazolyl)-2,5-diphenyl-2-H-tetrazolium bromide (MTT; Genview, Beijing, China) assay, following the manufacturer’s protocol. PANC-1 and SW1990 cells were inoculated into a 96-well plate at 100 µL/well volume (at a density of 2 × 10^3^ cells/pore). On the 1st, 2nd, 3rd, 4th, or 5th day, 20 µL of MTT solution (5 mg/mL) was added to the 96-well plates and the cell supernatant was removed after 4 h. Following this, 100 µL of dimethyl sulfoxide (DMSO) was added, and the plates were shaken at room temperature for 5 min. The optical density (OD) of each well was monitored at 490 nm using a microplate reader (M2009PR, Tecan infinite). All experiments were repeated at least three times, with the data expressed as mean ± standard deviation for statistical mapping.

### Flow cytometry for apoptotic assay

PANC-1 and SW1990 cells were seeded into 6-well plates at 2 mL/well volume and incubated for 5 days at 37 ^∘^C. Afterward, the cells were trypsinized, suspended and stained with Annexin V-allophycocyanin (V-APC) (eBioscience, San Diego, CA, USA) in the dark for 15 min. Finally, the cell phase percentages were determined using a flow cytometer (Millipore) to analyze the apoptotic rate.

### Transwell migration assay

According to the protocol provided in the literature, transwell experiments were performed to access the ability of cell migration [16]. PANC-1 and SW1990 cells (5 × 10^4^/well) were planted in 24-well plates and placed in the upper chamber of the transwell setup in serum-free culture medium. Meanwhile, 600 µL DMEM with a high concentration of FBS (30%) was supplemented in the lower chamber of the transwell. After 24 h, the cells that had adhered to the matrix glue of transwell were fixed in methanol for 30 min, stained with 0.1% crystal violet for 20 min, and then photographed under a 200× microscope in five randomly selected fields.

### Wound-healing assay

In order to determine the effect of CCNI2 on the migration of PANC-1 and SW1990, wound-healing experiments were performed as described above [17]. PANC-1 and SW1990 cells were seeded into a 96-well plate at a density of 1 × 10^5^ cells per well (100 µL/well) and cultured for 120 h. After the cells were treated with mitomycin C (Sigma M0503), scratches were generated using a scratch tester (VP science, VP408FH). Subsequently, the cells were rinsed in PBS for 3 times to remove the detached cells and then incubated in serum-free medium. Cells within the same field were identified at 0 h, 8 h, 24 h, 36 h, and 48 h, respectively, after migration, using the Cellomics (Thermo Fisher Scientific, Wilmington, DE, USA, ArrayScan VT1). The migration rate of each group was calculated based on the scratch distance using a fluorescence-based Cellomics ArrayScan VTI analyzer (Thermo Fisher Scientific). The migration ratio was calculated as [(S3+S4) − (S1+S2)] × 100%, where S1+S2 represents the wound area measured immediately after scratching (0 h), and S3+S4 represents the wound area measured at aforementioned hours after the scratch was performed.

### Human apoptosis antibody array

Experiments were conducted to assess the expression of apoptosis-related proteins, following the protocol provided by the Human Apoptosis Antibody Array kit (Abcam, Cat. #AB134001).

### Animal xenograft model

A suspension of 200 µL SW1990 cells (1×10^7^ cells/mL) was subcutaneously injected into the right forelimb axillary of four weeks old Bagg’s Albino/c (BALB/c) female nude mice (Shanghai Jiesijie Experimental Animal Co., Ltd., Shanghai, China). The mice were randomly divided into two groups: shCtrl (*n* ═ 5) vs shCCNI2 (*n* ═ 5). Following tumor formation, the mice’s body weight and tumor dimensions, its long and short diameters, were measured every 3 days using digital calipers, during the experimental period. Tumor volume was calculated as π/6×*L* ×*W* ×*W*, where *L* represents the long diameter and *W* represents the short diameter of the tumor. On the last day of breeding (22th day), the mice were anesthetized via intraperitoneal injection of 0.7% pentobarbital sodium at a dose of 10 µL/g. Tumor burden of the mice was assessed in a flat box under the multi-spectral living imaging system (Lumina LT, Perkin Elmer, MA, USA). All mice were subsequently euthanized by cervical dislocation, and tumor tissues were surgically excised. Before storage at −80 ^∘^C with liquid nitrogen, the tumor volumes were assessed with a caliper and the tumor weights were recorded. Finally, the tumor tissues were sectioned and subjected to immunohistochemical (IHC) experiments, as previously described, to examine the expression of the proliferation marker KI67 (1:200, Abcam, USA, Cat. # ab16667).

### Ethical statement

The research was approved by the Ethics Committee of Shanghai Tongji University. The experimental procedures were approved by the Ethics Committee of Hepatobiliary Surgery, the First Medical Center, Chinese PLA General Hospital. The animal experiments were approved by the Ethics Committee of the First Medical Center, Chinese PLA General Hospital, and were conducted in accordance with the guidelines and protocols for animal care and protection.

### Statistical analysis

All experiments were repeated independently at least three times, with the data expressed as mean ± standard deviation. Statistical differences between two groups were evaluated using the unpaired Student’s *t*-test. The data were analyzed by the GraphPad Prism 8.0 software (GraphPad Software Inc., San Diego, CA, USA) and the *P* values < 0.05 were considered statistically significant. The Mann–Whitney U test or Pearson correlation analysis was used to analyze the associations between CCNI2 expression and the clinicopathological features of pancreatic cancer. Survival curves were obtained by the Kaplan–Meier method, and differences in survival rates were assessed by the log-rank test. The qPCR was analyzed using the 2^−ΔΔCT^ method.

## Results

### CCNI2 is abundantly expressed in pancreatic cancer tissues and cell lines, and predicts poor prognosis

**Figure 1.**
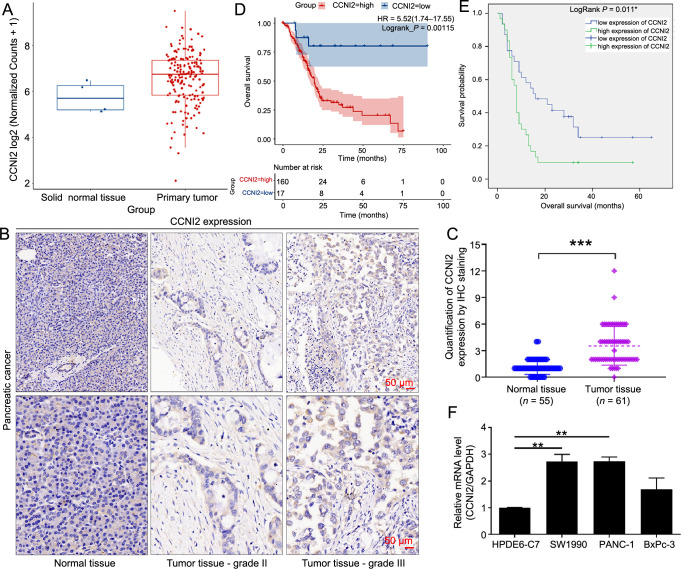
**Identification of the clinical correlation between CCNI2 and human pancreatic cancer**. (A) The expression of CCNI2 in normal pancreatic tissue and pancreatic cancer tissues, analyzed using the TCGA database; (B) Representative images of immunohistochemical staining showing the expression of CCNI2 in pancreatic cancer tissues and adjacent tissues; (C) Quantitative results of the immunohistochemical staining; (D) Log-rank test utilized to examine the difference in overall survival of pancreatic cancer patients between the high and the low CCNI2 expression groups; (E) A Kaplan–Meier analysis showing the association between CCNI2 expression levels and overall survival of pancreatic cancer patients; (F) The differences in relative mRNA expression of CCNI2 in pancreatic cancer cells PANC-1, SW1990, and BxPc-3 and normal pancreatic duct epithelial cells HPDE6-C7. Data are represented as mean ± SD. **P < 0.01, ***P < 0.001. CCNI2: Cyclin I-like protein; TCGA: The Cancer Genome Atlas; IHC: Immunohistochemistry; HR: Hazard ratio; *GAPDH*: Glyceraldehyde 3-phosphate dehydrogenase gene.

**Figure S1.**
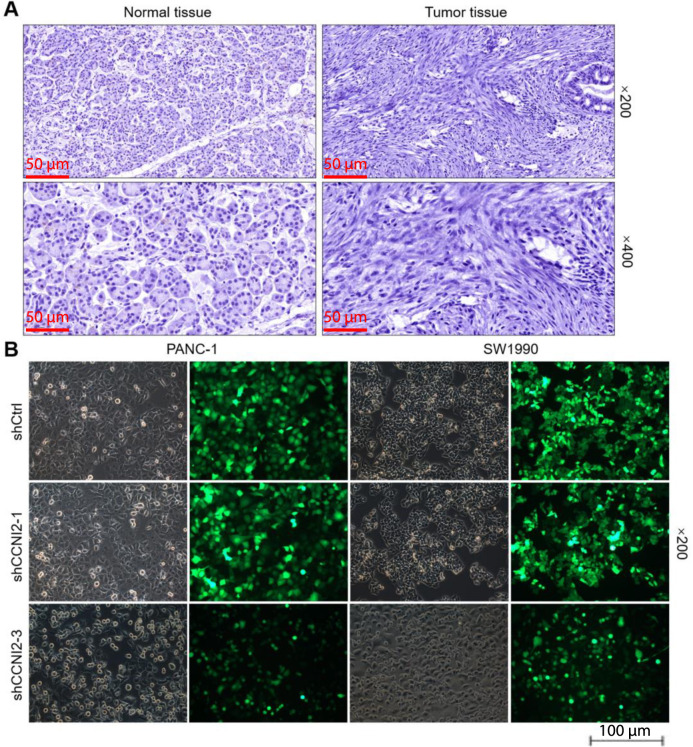
(A) Images of normal pancreatic tissue and tumor tissue after the IHC staining. The specificity of tumor and normal tissues were detected by IHC staining only after incubation with the secondary antibody. (B) Transfection efficiency of PANC-1 and SW1990 cells evaluated by the expression of GFP, 72 h post-infection. The presented results are representative of experiments repeated at least three times. IHC: Immunohistochemistry; GFP: Green fluorescent protein; shCtrl: Short hairpin control.

Firstly, the expression of CCNI2 in normal pancreatic tissue and pancreatic cancer tissues was analyzed using The Cancer Genome Atlas (TCGA) database, which indicated that CCNI2 was upregulated in pancreatic cancer (Figure 1A). To verify the correlation between CCNI2 expression and human pancreatic cancer, IHC staining was performed on tumor tissues and normal tissues from clinical pancreatic cancer patients (Figure 1B). Furthermore, a buffer solution with PBS, replacing the primary antibody, was used as a blank control for tissue staining. The staining results were negative, which confirmed the reliability of the results and ruled out non-specific color development caused by endogenous peroxidase and autofluorescence (Figure S1A). According to the quantitative results of IHC staining, scores higher than the median were defined as high CCNI2 expression, while scores below the median were defined as low CCNI2 expression (Figure 1C). Our data indicated that the expression of CCNI2 was significantly higher in pancreatic cancer tissues than in adjacent normal tissues (*P* < 0.001) (Table 1).

**Table 1:** Immunohistochemical analysis showing the CCNI2 expression pattern of pancreatic cancer tissues and adjacent tissues

**CCNI2 expression**	**Tumor tissue**		**Normal tissue**		***P* value**
	**Cases**	**Percentage**	**Cases**	**Percentage**	
Low	31	50.8%	53	96.4%	<0.0001
High	30	49.2%	2	3.6%	

After the obtained quantitative results of the IHC staining, scores higher than the median were defined as high CCNI2 expression, while scores below the median were defined as low CCNI2 expression. The expression of CCNI2 was significantly higher in pancreatic cancer tissues than in adjacent normal tissues (*P* < 0.001). CCNI2: Cyclin I-like protein; IHC: Immunohistochemistry.

Subsequently, the correlation between CCNI2 expression and the pathological features of pancreatic cancer was analyzed through clinical information. High expression of CCNI2 was observed in 10 out of 32 cases of low-grade pancreatic cancer (WHO I-II; 31.25%) and in 20 out of 29 cases of high-grade pancreatic cancer (WHO III; 68.97%) (*P* ═ 0.007) (Table 2). These findings suggest that increased CCNI2 expression was found in higher tumor grades. Consistently, a Pearson correlation analysis further established a significant correlation between CCNI2 expression and pathological characteristics of pancreatic cancer, such as pathological grade and pathological stage (Table 3).

**Table 2:** The relationship between CCNI2 expression and tumor characteristics in patients with pancreatic cancer

**Features**	**No. of patients**	**CCNI2 expression**		***P* value**
		**Low**	**High**	
**All patients**	61	31	30	
**Age (years)**				0.254
<67	30	13	17	
≥67	31	18	13	
**Sex**				0.879
Male	36	18	18	
Female	25	13	12	
**Number of positive lymph nodes**				0.520
≤0	33	18	15	
>0	24	11	13	
**Tumor size**				0.870
<4 cm	23	12	11	
≥4 cm	38	19	19	
**Grade**				0.007
I	1	0	1	
II	31	22	9	
III	29	9	20	
**Stage**				0.021
1	5	5	0	
2	43	22	21	
4	4	1	3	
**T category**				0.163
T1	2	1	1	
T2	7	6	1	
T3	36	18	18	
**Lymphatic metastasis**				0.624
N0	33	18	15	
N1	25	12	13	

The correlation between CCNI2 expression and the pathological features of pancreatic cancer was analyzed through clinical information. High expression of CCNI2 was observed in 10 out of 32 cases of low-grade pancreatic cancer (WHO I-II: 31; 25%) and in 20 out of 29 cases of high-grade pancreatic cancer (WHO III: 68; 97%) (*P* ═ 0.007). These findings suggest that increased CCNI2 expression was found in higher tumor grades. CCNI2: Cyclin I-like protein.

**Table 3:** The relationship between CCNI2 expression and tumor characteristics in patients with pancreatic cancer

		**CCNI2**
Grade	Pearson correlation	0.350
	Significance (double-tail)	0.006
	N	61
Stage	Pearson correlation	0.322
	Significance (double-tail)	0.020
	N	52

A Pearson correlation analysis established a significant correlation between CCNI2 expression and pathological characteristics of pancreatic cancer, such as pathological grade and pathological stage. CCNI2: Cyclin I-like protein.

Meanwhile, a log-rank test was used to examine the difference in overall survival of pancreatic cancer patients between high and low CCNI2 expression groups, based on data from the TCGA database. The results showed that the overall survival time of the high CCNI2 expression group was significantly shorter than that of the low CCNI2 expression group (*P* ═ 0.00115) (Figure 1D). Consistently, the clinical relevance of CCNI2 in the human pancreatic cancer was verified through a Kaplan–Meier survival analysis, suggesting that higher expression of CCNI2 is indicative of a worse prognosis (*P* ═ 0.011) (Figure 1E). Collectively, these findings demonstrate that CCNI2 was highly expressed in pancreatic cancer and was associated with poor prognosis.

### CCNI2 is knocked down and amplified in lentivirus-mediated pancreatic cancer cells

**Figure 2.**
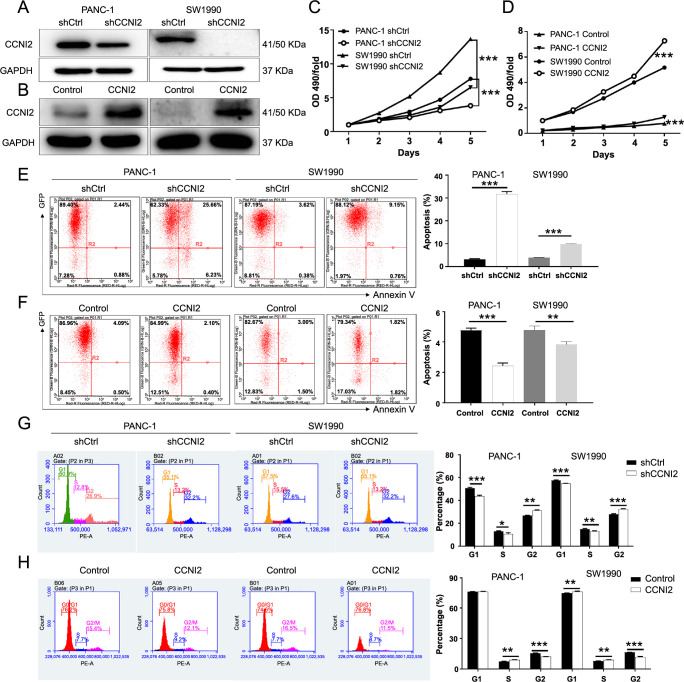
**CCNI2 is required for the proliferation, apoptosis, and cell cycle progression of pancreatic cancer cells**. (A and B) Models of CCNI2 knockdown and overexpression constructed in PANC-1 and SW1990 cells, respectively; (C and D) The cell growth curves based on the MTT assay which was used to detect the changes in the proliferation ability of PANC-1 and SW1990 cells after CCNI2 knockdown (C)/overexpression (D); (E and F) Flow cytometry analysis based on Annexin V-APC staining utilized to detect the percentage of early apoptotic cell for PANC-1 and SW1990 cells, after CCNI2 knockdown (E)/overexpression (F); (G and H) The effects of downregulation (G) and upregulation (H) of CCNI2 on the cell cycle phase distribution of PANC-1 and SW1990, detected by flow cytometry. The presented results are representative of experiments repeated at least three times. Data are represented as mean ± SD. *P < 0.05, **P < 0.01, ***P < 0.001. CCNI2: Cyclin I-like protein; *GAPDH*: Glyceraldehyde 3-phosphate dehydrogenase gene; shCtrl: Short hairpin control; Annexin V-APC: Annexin V-allophycocyanin; OD: Optical density.

**Figure S2.**
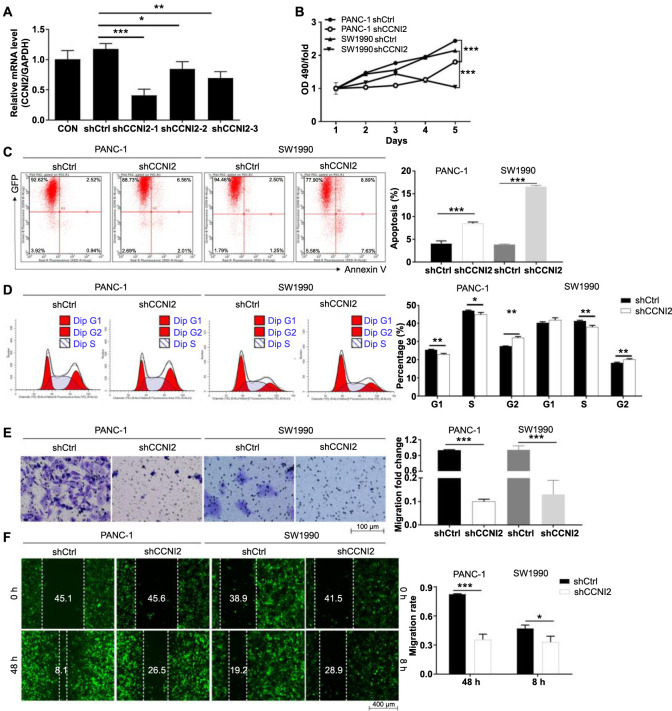
(A) The differences in relative mRNA expression of CCNI2 in shCCNI2-1, shCCNI2-2, and shCCNI2-3 sequences screened by qPCR. (B–F) Changes of the proliferation (B), apoptosis (C), cell cycle progression (D), and migration (E and F) of PANC-1 and SW1990 cells, after the CCNI2 knockdown. The results are obtained by repeating three independent experiments. Data are represented as mean ± SD. *P < 0.05, **P < 0.01, ***P < 0.001. CCNI2: Cyclin I-like protein; *GAPDH*: Glyceraldehyde 3-phosphate dehydrogenase gene; shCtrl: Short hairpin control; qPCR: Quantitative PCR; OD: Optical density.

The expression level of CCNI2 in PANC-1 and SW1990 cells was found to be significantly higher than that in normal pancreatic ductal epithelial cells HPDE6-C7 (*P* < 0.01) (Figure 1F). To investigate the function of CCNI2 in pancreatic cancer, models of CCNI2 knockdown and overexpression were constructed in PANC-1 and SW1990 cells, respectively. The successful transduction of lentivirus shCtrl, shCCNI2-1, and shCCNI2-3 into pancreatic cancer cells was confirmed by the high expression of GFP in PANC-1 and SW1990 cells (Figure S1B). To identify the most effective shRNA sequences against CCNI2, three shRNA sequences were constructed and their knockdown efficiency was compared with the control group (shCtrl). The mRNA expression level of CCNI2 was significantly lower in the shCCNI2-1 and shCCNI2-3 sequences, thus these two targets were selected for downstream functional testing experiments and were collectively referred to as shCCNI2 (Figure S2A). Furthermore, the protein level of CCNI2 in the shCCNI2 group was downregulated compared to the shCtrl group (Figure 2A). Conversely, the protein level of CCNI2 was upregulated in the CCNI2 group compared to the control group, indicating that CCNI2 was amplified in PANC-1 and SW1990 cells (Figure 2B).

**Figure 3.**
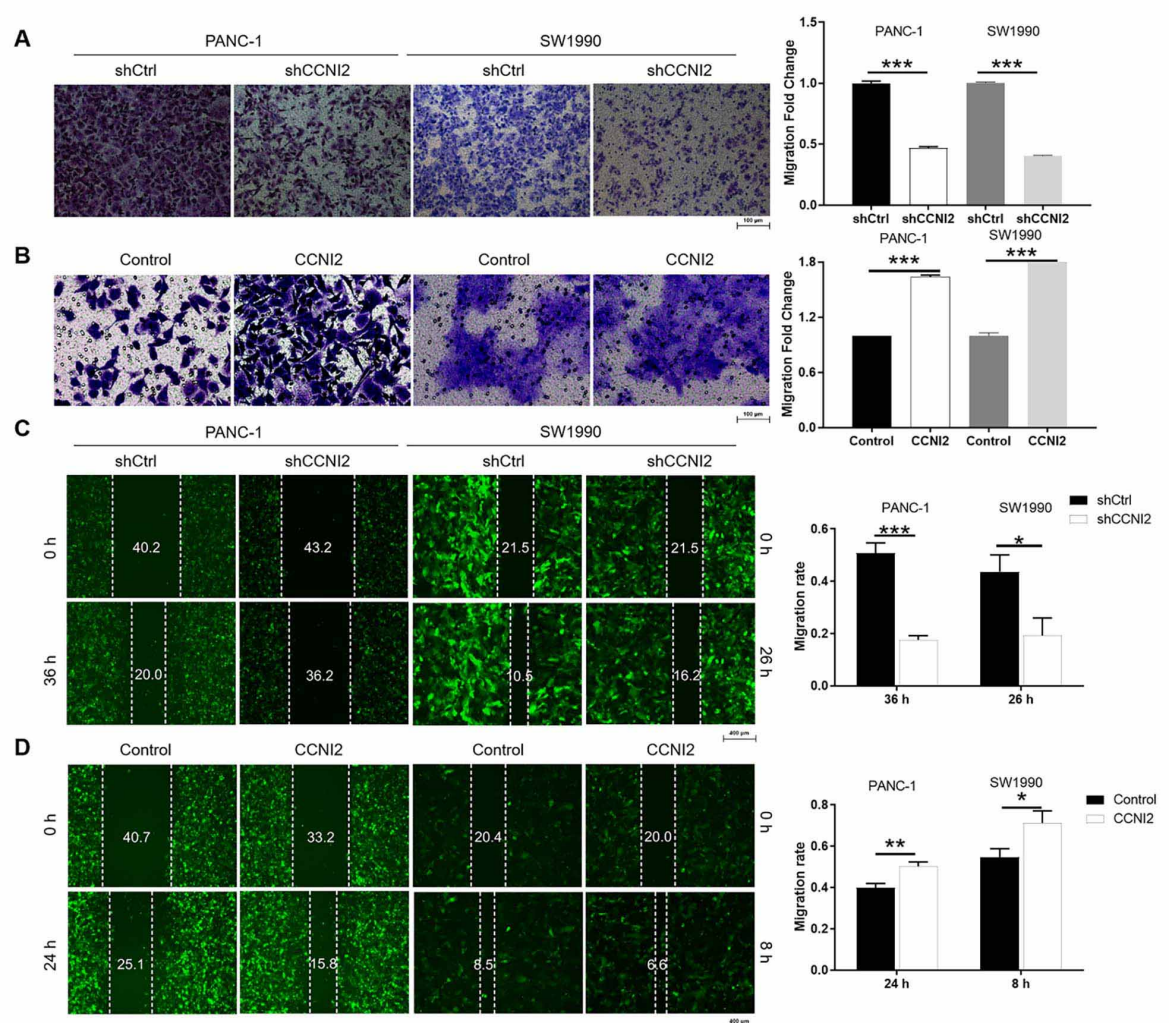
**CCNI2 is required for the migration of pancreatic cancer cells in vitro**. (A and B) The effects of downregulation (A) and upregulation (B) of CCNI2 on PANC-1 and SW1990 cell migration, detected by the transwell assay; (C and D) The migration of PANC-1 and SW1990 cells with the downregulation (C) and upregulation (D) of CCNI2, evaluated by the wound-healing assay. The results are obtained by repeating three independent experiments. Data are represented as mean ± SD. *P < 0.05, **P < 0.01, ***P < 0.001. CCNI2: Cyclin I-like protein; shCtrl: Short hairpin control.

### CCNI2 is required for the proliferation, apoptosis, and cell cycle progression of pancreatic cancer cells

The biological effects of CCNI2 in human pancreatic cancer cells PANC-1 and SW1990 were determined through a series of in vitro loss/gain-of-function experiments. As shown in Figure 2C, cell growth curves based on the MTT assay demonstrated a significant decrease in the proliferation ability of PANC-1 and SW1990 cells after CCNI2 knockdown (*P* < 0.001). Consistently, another shCCNI2 target showed similar inhibitory effects on the proliferation of PANC-1 and SW1990 cells (*P* < 0.001) (Figure S2B). Conversely, the proliferation of PANC-1 and SW1990 cells was significantly enhanced following the CCNI amplification (*P* < 0.001) (Figure 2D). Furthermore, the effects of CCNI2 knockdown and overexpression on apoptosis of pancreatic cancer cells were analyzed by flow cytometry with Annexin V staining. The percentage of apoptotic PANC-1 and SW1990 cells in the shCCNI2-1 group was higher compared to the shCtrl group (*P* < 0.001) (Figure 2E). As expected, the same trend was observed in PANC-1 and SW1990 cells with another shCCNI2 target knockdown (*P* < 0.001) (Figure S2C). Conversely, the apoptosis rate of pancreatic cancer cells decreased after CCNI2 overexpression (*P* < 0.01) (Figure 2F). Moreover, our data indicated that CCNI2 had an effect on the cell cycle progression of pancreatic cancer cells. The CCNI2 knockdown cells showed a decrease in the S phase and an increase in the G2 phase of the cell cycle, while the cells after CCNI2 overexpression showed the opposite trend (Figure 2G and 2H and Figure S2D). Therefore, CCNI2 was found to be necessary for the proliferation, apoptosis, and cell cycle progression of pancreatic cancer cells.

**Figure 4.**
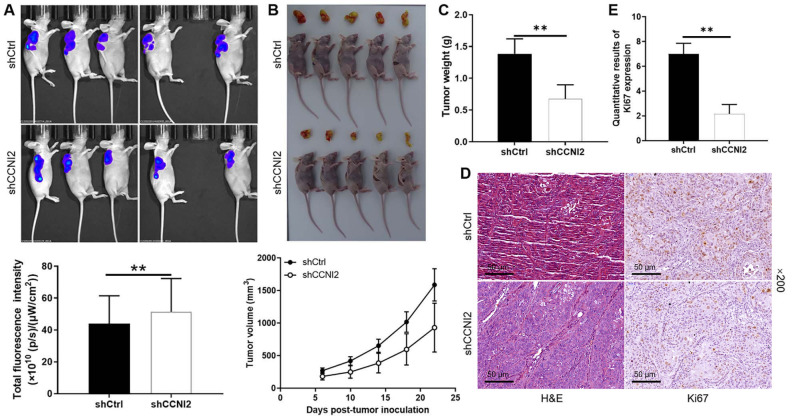
**CCNI2 knockdown attenuates tumor growth in mice xenograft models**. (A) Detection of the fluorescence intensity of mice tumors in the shCCNI2 group and the shCtrl group under in vivo imaging system on day 22, after PANC-1 cells with reduced CCNI2 expression were subcutaneously injected into the mice; (B) The mice were monitored for 22 days, the average tumor volume of mice in the shCCNI2 group and the shCtrl group was measured and the tumor was photographed; (C) The average evaluated tumor weights in the shCtrl group and the shCCNI2 group; (D) Hematoxylin and eosin staining and IHC (detected Ki67 expression) performed on tumor tissues in the shCtrl group and the shCCNI2 group, respectively; (E) Quantitative results of Ki67 expression. Data are represented as mean ± SD. *P < 0.05, **P < 0.01, ***P < 0.001. CCNI2: Cyclin I-like protein; shCtrl: Short hairpin control; IHC: Immunohistochemistry; H&E: Hematoxylin and eosin.

**Figure 5.**
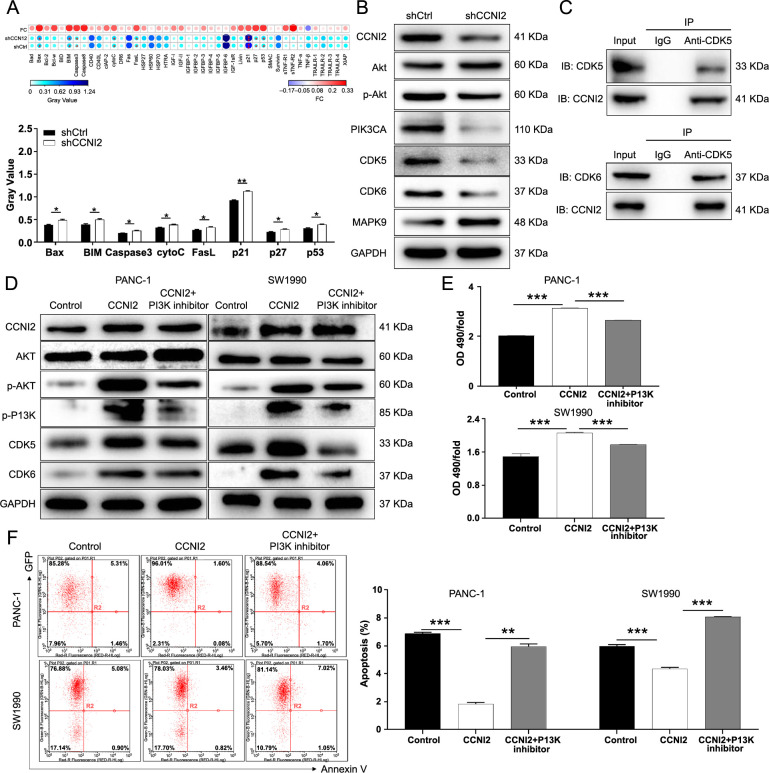
**Effects of CCNI2 knockdown on apoptosis-related proteins and typical signal pathways in pancreatic cancer cells**. (A) PANC-1 cells with knocked down CCNI2 underwent analysis using a human apoptosis antibody array to examine the expression of apoptosis-related proteins. The results were analyzed based on the gray value analysis of these two spots, and this analysis was repeated three times. (B) Western blot analysis examining the protein expression of key components in typical signaling pathways within PANC-1 cells where CCNI2 was knocked down. (C) Protein interaction between CCNI2 and CDK5/6 confirmed by Co-IP. (D) Pancreatic cancer cells overexpressing CCNI2 treated with PI3K inhibitors to detect changes in proteins associated with typical signaling pathways. (E and F) PI3K inhibitors were used to treat pancreatic cancer cells with CCNI2 overexpression, and the proliferation (E) and apoptosis (F) were detected. The results are obtained by repeating three independent experiments. Data are represented as mean ± SD. *P < 0.05, **P < 0.01, ***P < 0.001. CCNI2: Cyclin I-like protein; shCtrl: Short hairpin control; Bax: Bcl-2-associated X protein; BIM: Bcl-2 interacting mediator of cell death; Caspase3: Cysteine-aspartic proteases 3; cytoC: Cytochrome C; FasL: Fas ligand; p21: Cyclin-dependent kinase inhibitor 1A; p27: Cyclin-dependent kinase inhibitor 1B; AKT: Protein kinase B; p-AKT: Phosphorylated-protein kinase B; PIK3CA: Phosphatidylinositol-4,5-bisphosphate 3-kinase catalytic subunit alpha; CDK5: Cycin-dependent kinase 5; CDK6: Cycin-dependent kinase 6; MAPK9: Mitogen-activated protein kinase 9; GAPDH: Glyceraldehyde 3-phosphate dehydrogenase; IB: Immunoblotting; IP: Immunoprecipitation; Co-IP: Co-immunoprecipitation; IgG: Immunoglobulin G; PI3K: Phosphatidylinositol 3-kinase; p-PI3K: Phosphorylated-phosphatidylinositol 3-kinase; OD: Optical density; Bad: Bcl-2-associated death promoter; Bcl-2: B-cell lymphoma 2; Bcl-w: B-cell lymphoma w; BID: BH3 interacting-domain death agonist; Caspase8: Cysteine-aspartic protease 8; CD40: Cluster of differentiation 40; CD40L: CD40 ligand; cIAP-2: Cellular inhibitor of apoptosis protein 2; DR6: Death receptor 6; HSP: Heat shock protein; HTRA: High-temperature requirement A; IGF-I: Insulin-like growth factor I; IGF-II: Insulin-like growth factor II; IGFBP: Insulin-like growth factor-binding protein; IGF-1sR: Insulin-like growth factor 1 soluble receptor; SMAC: Second mitochondria-derived activator of caspases; sTNF-R1: Soluble tumor necrosis factor receptor 1; sTNF-R2: Soluble tumor necrosis factor receptor 2; TNF-a: Tumor necrosis factor alpha; TNF-ß: Tumor necrosis factor beta; TRAILR: TNF-related apoptosis-inducing ligand receptor; XIAP: X-linked inhibitor of apoptosis protein.

### CCNI2 drives the migration of pancreatic cancer cells in vitro

Furthermore, the migration ability of pancreatic cancer cells in response to alterations in CCNI2 expression was examined. Cells that migrated in the shCCNI2 and shCtrl groups were stained, photographed, and counted from a randomly selected microscopic field of view. The results of the transwell assays indicated a significant reduction in the number of migratory PANC-1 and SW1990 cells in the shCCNI2 groups compared to the shCtrl group (*P* < 0.001) (Figure 3A and Figure S2E). Moreover, compared with the control group, more cells in the CCNI2 overexpression group were stained, indicating that the CCNI2 overexpression could promote the migration of pancreatic cancer cells (*P* < 0.001) (Figure 3B). To further determine the effect of CCNI2 on the migration of PANC-1 and SW1990 cells, wound-healing experiments were conducted. As anticipated, the results from the two sets of targeting sequences consistently showed a significantly lower migration rate of PANC-1 and SW1990 cells in the shCCNI2 group compared to the shCtrl group after CCNI2 knockdown (*P* < 0.05) (Figure 3C and Figure S2E). Additionally, PANC-1 and SW1990 cells overexpressing CCNI2 exhibited a stronger migration ability compared to the control group (*P* < 0.05) (Figure 3D). In conclusion, CCNI2 can accelerate the migration ability of PANC-1 and SW1990 cells.

### CCNI2 knockdown impairs tumorigenesis in the mice xenograft model

Although in vitro experiments have shown that CCNI2 contributes to the malignant phenotypes of pancreatic cancer cells, further investigation is needed to explore its role in vivo. Therefore, PANC-1 cells with decreased CCNI2 expression were subcutaneously injected into nude mice to establish a xenograft model. As illustrated in Figure 4A, the fluorescence intensity of the tumors in the shCCNI2 group was significantly weaker than that in the shCtrl group, indicating that the tumorigenesis ability of PANC-1 cells was reduced after CCNI2 knockdown (*P* < 0.01). The data collected over a 22-day period of monitoring showed that the average volume of tumors formed by cells in the CCNI2 silenced group was significantly smaller compared to that in the negative control group (*P* < 0.01) (Figure 4B). Similarly, the average tumor weight in the shCCNI2 group was 0.676 ± 0.222 g, which was lower than that in the shCtrl group (*P* < 0.01) (Figure 4C). Furthermore, hematoxylin and eosin (H&E) staining was performed to confirm that the nodules developed in mice were indeed tumors, by observing the tissue structure and the number of nuclei. The results from the IHC staining showed that the Ki67 expression in xenograft tumors, which developed from the CCNI2 knocked down PANC-1 cells, was significantly downregulated compared to tumors derived from shCtrl cells (Figure 4D and 4E). Overall, the reduction of CCNI2 expression impaired tumor formation in mice, confirming the validity of the in vitro study findings.

### CCNI2 promotes pancreatic cancer through the phosphatidylinositol 3-kinase (PI3K)/protein kinase B (AKT) signaling pathway

To investigate the effect of CCNI2 on the expression of apoptosis-related proteins in pancreatic cancer cells, a human apoptosis antibody array was employed for comparing PANC-1 cells in the shCCNI2 and shCtrl groups. Among the 43 proteins analyzed, knockdown of CCNI2 upregulated the expression levels of pro-apoptotic proteins, such as bcl-2-associated X protein (Bax), bcl-2 interacting mediator of cell death (BIM), cysteine-aspartic proteases 3 (Caspase3), cytochrome C (cytoC), fas ligand (FasL), cyclin-dependent kinase inhibitor 1A (p21), cyclin-dependent kinase inhibitor 1B (p27), and p53 (*P* < 0.05) (Figure 5A). Additionally, the study demonstrated that CCNI2 knockdown led to decreased downregulation of CDK5 and cyclin-dependent kinase 6 (CDK6), while promoting the production of mitogen-activated protein kinase 9 (MAPK9) (Figure 5B). It has been previously reported that CCNI2 interacts with CDK5 to activate the kinase activity of CDK5 and participate in cell cycle regulation [9]. Through co-immunoprecipitation (Co-IP), we found evidence of a protein interaction between CCNI2 and CDK5/6 in pancreatic cancer cells (Figure 5C).

Furthermore, the PI3K/AKT signaling pathway is one of the most common pathways found in human tumors. This pathway plays a role in various cancer-related behaviors, including cell survival, proliferation, growth, apoptosis, and metastasis [18]. Our data suggests that knocking down CCNI2 can reduce the protein expression of key components in the PI3K/AKT pathway. Additionally, the levels of AKT and PI3K phosphorylation are increased in pancreatic cancer cells that overexpress CCNI2, and treating these cells with a PI3K inhibitor can partially reverse this effect (Figure 5D). Moreover, inhibition of this pathway partially mitigates the proliferative (promoting) and apoptotic (inhibiting) effects of CCNI2 in pancreatic cancer cells (Figure 5E and 5F). Although the exact mechanism is still unclear, preliminary findings suggest that CCNI2 may regulate pancreatic cancer through the PI3K/AKT signaling pathway.

## Discussion

Globally, pancreatic cancer is recognized as one of the deadliest malignancies, lacking effective targeted therapies. Previous studies have shown that the prognosis of pancreatic cancer is remarkably influenced by the size of the tumor, T cell infiltration, lymphatic infiltration, histological lymph node involvement, and metastasis [19, 20]. To explore the factors that influence the prognosis of this disease, we targeted the role of CCNI2, a cycle-associated protein, in pancreatic cancer. Our data identified abnormally high expression of CCNI2 in human pancreatic cancer. In addition, the expression of CCNI2 was positively correlated with the pathological grade and pathological stage. Moreover, pancreatic cancer patients with high CCNI2 expression usually had a shorter survival time, which may be an indicator of poor prognosis.

Furthermore, our results demonstrated significant inhibition of malignant behaviors in CCNI2 knockdown pancreatic cancer cells. This was characterized by weakened proliferation, impeded migration, and enhanced apoptosis. It has been proven that inducing tumor cell apoptosis is a key strategy in developing innovative drugs for cancer treatment [21]. Therefore, it is necessary to explore the mechanisms related to apoptosis for effective cancer treatment [22]. Given the poor efficacy and severe toxicity of current standard chemotherapy for pancreatic cancer, there is a growing interest in developing new treatment methods that induce apoptosis [21]. The present study found that the knockdown of CCNI2 promoted apoptosis in pancreatic cancer cells by activating the expression of pro-apoptotic proteins. These proteins include Bax, BIM, Caspase3, cytoC, FasL, p21, p27, and p53. Previous studies have shown that these apoptosis-related proteins are important regulators of cell proliferation and apoptosis. They can activate both endogenous and exogenous apoptosis pathways, leading to apoptosis [23–25]. Therefore, we speculate that the knockdown of CCNI2 promotes the apoptosis process in pancreatic cancer cells through a series of cascades involving apoptosis-related proteins.

Previous studies have reported that the activation of the PI3K/AKT pathway contributes to the occurrence and development of pancreatic cancer [26]. For example, Bondar et al. [27] suggested that inhibiting the PI3K/AKT pathway induces apoptosis and reduces the proliferation of pancreatic cancer cells. Similarly, Mao et al. [28] found that inhibiting the PI3K/AKT pathway could potentially serve as a therapy for pancreatic cancer. In this study, our results demonstrate that the knockdown of CCNI2 leads to a decrease in the AKT phosphorylation and downregulation of phosphadidylinositol-4,5-bisphosphate 3-kinase catalytic subunit alpha (PIK3CA). Additionally, the phosphorylation levels of AKT and PI3K in pancreatic cancer cells overexpressing CCNI2 were increased, and treatment with a PI3K inhibitor partially reversed this effect. Furthermore, treatment with a PI3K inhibitor attenuated the promotion of CCNI2 overexpression on pancreatic cancer cells. Therefore, it can be inferred that CCNI2 may regulate the proliferation and apoptosis of pancreatic cancer through the PI3K/AKT signaling pathway.

As a novel activator of CDK5, CCNI2 has been proven to physically interact with CDK5 and activate its kinase activity [9]. This study demonstrates that knockdown/overexpression of CCNI2 in pancreatic cancer cells can downregulate/upregulate the expression of CDK5 and CDK6. Moreover, as a member of the cell cycle-regulated kinase family, CDK6 is an important and actionable target in pancreatic cancer [29]. Our data indicate that CCNI2 has an effect on the cell cycle progression of pancreatic cancer cells. CCNI2 knocked down cells showed a decrease in the S phase and an increase in the G2 phase, while cells with CCNI2 overexpression displayed the opposite effect. However, these data are only preliminary investigations, and the specific mechanism of how CCNI2 affects the cell cycle warrants further in-depth study.

As a typical component of the MAPK signaling pathway, MAPK9 plays an important role in tumorigenesis [30, 31]. This pathway is involved in the migration, invasion, and angiogenesis of pancreatic cancer by regulating tubulin aggregation [32]. In this study, the knockdown of CCNI2 led to the upregulation of MAPK9 in pancreatic cancer cells. This preliminary detection of the influence of CCNI2 on MAPK9 lays the foundation for further exploration in the future.

## Conclusion

The abnormally high expression of CCNI2 in pancreatic cancer holds clinical value in predicting a poor prognosis. Moreover, CCNI2 plays a crucial role in promoting the proliferation, apoptosis, cell cycle progression, and migration of tumor cells, thereby driving the progression of pancreatic cancer. Consequently, the established upregulation of CCNI2 in pancreatic cancer highlights its potential as a prognostic marker and therapeutic target for this disease.

## Supplemental data

## Data Availability

The data used to support the findings of this study are available from the corresponding author upon request.
